# Distribution of static and dynamic cyclotorsion and influencing factors in FS-LASIK

**DOI:** 10.3389/fmed.2025.1614717

**Published:** 2026-01-30

**Authors:** Jiliang Ning, Yanan Mu, Siyu Sun, Taorui Yu, Xiaoyu Liu, Qiaosi Zhang, Lijun Zhang

**Affiliations:** 1 Department of Ophthalmology, The Third People’s Hospital of Dalian, Dalian, China; 2 Department of Ophthalmology, Dalian Municipal Eye Hospital, Dalian, China; 3 Liaoning Provincial Key Laboratory of Cornea and Ocular Surface Diseases, Dalian, China; 4 Liaoning Provincial Optometry Technology Engineering Research Center, Dalian, China

**Keywords:** corneal surgery, laser, dynamic cyclotorsion, keratomileusis, laser *in situ*, static cyclotorsion

## Abstract

**Objective:**

This study evaluated the static cyclotorsion component (SCC) and dynamic cyclotorsion component (DCC) during femtosecond laser-assisted stromal *in situ* keratomileusis (FS-LASIK) and identified factors influencing these components.

**Methods:**

This retrospective cross-sectional study included 196 patients (392 eyes) with refractive errors who underwent bilateral FS-LASIK. The cohort comprised 105 men and 91 women (mean age: 23.88 ± 6.90 years). An IntraLase femtosecond laser machine was used to create the corneal flap, whereas an AMARIS excimer laser machine was used to ablate the corneal stroma. Parameters related to SCC and DCC were collected using an eye tracker, which also calculated the registration success rate. Preoperative and intraoperative variables were analyzed.

**Results:**

Static cyclotorsion was successfully registered in 80.6% of eyes, with corneal flap thickness [odds ratio (OR) = 0.903, *p* < 0.01] and laser cavity temperature (OR = 1.26, *p* = 0.047) identified as significant factors associated with registration failure. No significant correlation was found between absolute SCC and preoperative or intraoperative variables (all *p* > 0.05). For DCC, 98.7% of eyes were successfully registered, with a median amplitude of 1.00 degrees (0.78–1.30 degrees). Spearman’s correlation analysis revealed significant correlations between age, equivalent spherical power, laser cavity temperature, actual ablation time, and DCC amplitude (all *p* < 0.05).

**Conclusion:**

Thin corneal flap and elevated laser cavity temperature are risk factors associated with SCC registration failure. Age, equivalent spherical power, laser cavity temperature, and actual ablation time were correlated with dynamic cyclotorsion, highlighting the need for precise tracking in FS-LASIK.

## 1 Introduction

Laser corneal refractive surgery corrects myopia by reshaping the cornea using a laser, thereby altering its refractive power. Laser-assisted stromal *in situ* keratomileusis (LASIK) is a well-established surgical technique that provides favorable outcomes and high predictability in correcting astigmatism (1). Cyclotorsion refers to the rotational movement of the eyeball around the visual axis and is classified into two types: static and dynamic. The static cyclotorsion component (SCC) represents eye rotation induced by the vestibular system when the body shifts from a sitting to a lying position (2, 3). Conversely, the dynamic cyclotorsion component (DCC) denotes eye rotation induced by mental stress during surgical ablation (4). During the procedure, unavoidable eye rotation and other factors may cause the laser ablation position to deviate from the intended direction, resulting in varying degrees of astigmatism and postoperative higher-order aberrations (5–8). The current AMARIS excimer laser system (SCHWIND eye-tech-solutions, Kleinostheim, Germany) incorporates advanced SCC and DCC compensation mechanisms to enhance refractive outcomes (9, 10). However, challenges remain regarding the cyclotorsion compensation registration success rate and compensation lag. This study is novel in its comprehensive evaluation of SCC and DCC during femtosecond LASIK (FS-LASIK), providing new insights into how these factors affect surgical outcomes. Additionally, we investigate the influence of various preoperative and intraoperative parameters on these cyclotorsion components and identify factors associated with the success rate of cyclotorsion tracking registration.

## 2 Materials and methods

### 2.1 Research subjects

This retrospective cross-sectional study analyzed the medical records of 196 patients (392 eyes) with refractive errors who underwent bilateral FS-LASIK at the Refractive Surgery Center of Dalian Third People’s Hospital between January 2023 and November 2024. All enrolled patients were of Han Chinese ethnicity. The cohort included 105 men (210 eyes) and 91 women (182 eyes), with a mean age of 23.88 ± 6.90 years (range: 17–52 years). Inclusion criteria were as follows: stable refractive error for at least 2 years; preoperative best-corrected visual acuity (BCVA, measured in decimal notation) > 0.8; discontinuation of soft contact lens wear for at least 1 week, rigid contact lens wear for at least 1 month, and orthokeratology lens wear for at least 3 months before surgery. All patients underwent simultaneous FS-LASIK in both eyes. Exclusion criteria included nystagmus, neurological disorders affecting eye movement (e.g., vestibular disease), keratoconus or suspected keratoconus, other forms of corneal ectasia, active ocular disease, and mental health symptoms (e.g., anxiety and depression). This study adhered to the principles of the Declaration of Helsinki and was approved by the hospital’s ethics committee (approval number: 2024-166-001). A waiver of informed consent was granted in accordance with the requirements of the ethics committee.

### 2.2 Method of examination

Preoperative data, including age, sex, corneal curvature, equivalent spherical power, spherical power, cylindrical power, and pupil offset, were recorded. Intraoperative observation parameters included SCC and absolute SCC, as well as the maximum, minimum, average, and range of DCC. Additional variables included the diameters of the main optical and transition zones, central ablation depth, average laser cavity temperature and humidity, and theoretical and actual ablation times.

### 2.3 Ablation procedure and torsional data

All surgeries were performed by a single surgeon. Antibiotic eye drops were routinely administered for 3 days before surgery to prevent infection. The conjunctival sac was rinsed, and an IntraLase iFS150 femtosecond laser machine (Abbott Medical Company, United States) was used to create the flap after topical anesthesia. The corneal flap thickness was set at 90–130 μm with a 9.0 mm diameter. After lifting the corneal flap, an AMARIS 750S excimer laser (Schwind Company, Germany) was used in aberration-free mode for corneal stromal ablation. The main optical zone diameter was set between 5.5 and 7.0 mm. Following ablation, the corneal stromal bed was irrigated with a balanced salt solution, and the corneal flap was repositioned.

Iris images were obtained on the day of surgery by the same experienced technician. Before laser activation, the eye tracker captured an image of the iris with the patient in the supine position. This image was compared with the iris image obtained in the upright position before surgery to assess SCC. If iris registration fails, skip SCC and proceed with conventional surgery. Repeated intraoperative images were compared with the initial pre-ablation image to evaluate DCC. The eye tracker output included SCC values and the minimum, maximum, and average DCC values. Negative SCC and DCC values indicated clockwise rotation, whereas positive values indicated counterclockwise rotation. Incyclotorsion was defined as positive for the right eye and negative for the left eye, with the opposite designation for excyclotorsion. The DCC amplitude was calculated as the difference between the maximum and minimum DCC values.

### 2.4 Statistical analysis

Statistical analyses and image processing were performed using SPSS software (version 26.0, Chicago, IL, United States) and GraphPad Prism software (version 10.0, Boston, MA, United States). The Kolmogorov–Smirnov test was used to assess data normality. Normally distributed data were expressed as mean ± standard deviation (X ± S), whereas non-normally distributed data were expressed as the median (interquartile range) [M (Q1, Q3)]. The upper and lower bounds for all parameters were also provided. Multivariate logistic analysis was performed to evaluate the relationship between successful identification of static cyclotorsion and various preoperative and intraoperative variables. Additionally, Spearman’s correlation analysis was used to assess associations between absolute SCC and DCC amplitude and the preoperative and intraoperative variables. Scatter plots were generated for statistically significant results, with *p* < 0.05 considered statistically significant.

## 3 Results

This study included 196 patients, comprising 105 men and 91 women, with a mean age of 23.88 ± 6.90 years. The mean preoperative spherical equivalent was −7.21 ± 1.83 D, and the mean cylindrical power was −2.15 ± 0.99 D. The mean intraoperative optical zone diameter was 6.18 ± 0.29 mm, the mean maximum ablation depth was 116.14 ± 15.30 μm, and the mean actual ablation time was 24.68 ± 11.46 s. The mean temperature within the laser cavity was 24.30 ± 1.22°C, and the mean humidity was 36.46 ± 6.87%. Further details are presented in Table 1.

**TABLE 1:** Demographic, ophthalmic, and intraoperative parameters of eyes.

Parameter	Mean ± SD	Range
Number of eyes (R/L)	392(196/196)	
Sex (male/female)	105/91	
Age, years	23.88 ± 6.90	17–52
CCT, μm	546.64 ± 24.98	492–632
K1, D	42.40 ± 1.35	39.20–46.31
K2, D	44.78 ± 1.52	40.58–49.38
Km, D	43.59 ± 1.37	40.05–47.16
Max ablation depth, μm	116.14 ± 15.30	39–146
Ablation volume, nL	2527.43 ± 302.85	1,208–3,445
Sphere, D	−6.12 ± 2.02	−10.75–1.00
Astigmatism, D	−2.15 ± 0.99	−6.00–0
spherical equivalent, D	−7.21 ± 1.83	−11.88 to 1.00
Optical zone, mm	6.18 ± 0.29	5.50-7.00
Transition zone, mm	1.68 ± 0.20	0.85-2.00
Temperature-mean,°C	24.30 ± 1.22	20.75–28.10
Humidity-mean,%	36.46 ± 6.87	26.0-65.5
TreatmentDuration_theory, sec	18.17 ± 2.10	9–24
Ablation time, sec	24.68 ± 11.46	9–112
Pupil offset, μm	176.81 ± 88.89	0-490.29

CCT, central corneal thickness; K1, flat corneal keratometry; K2, steep corneal keratometry; Km, mean corneal keratometry.

**FIGURE 1:**
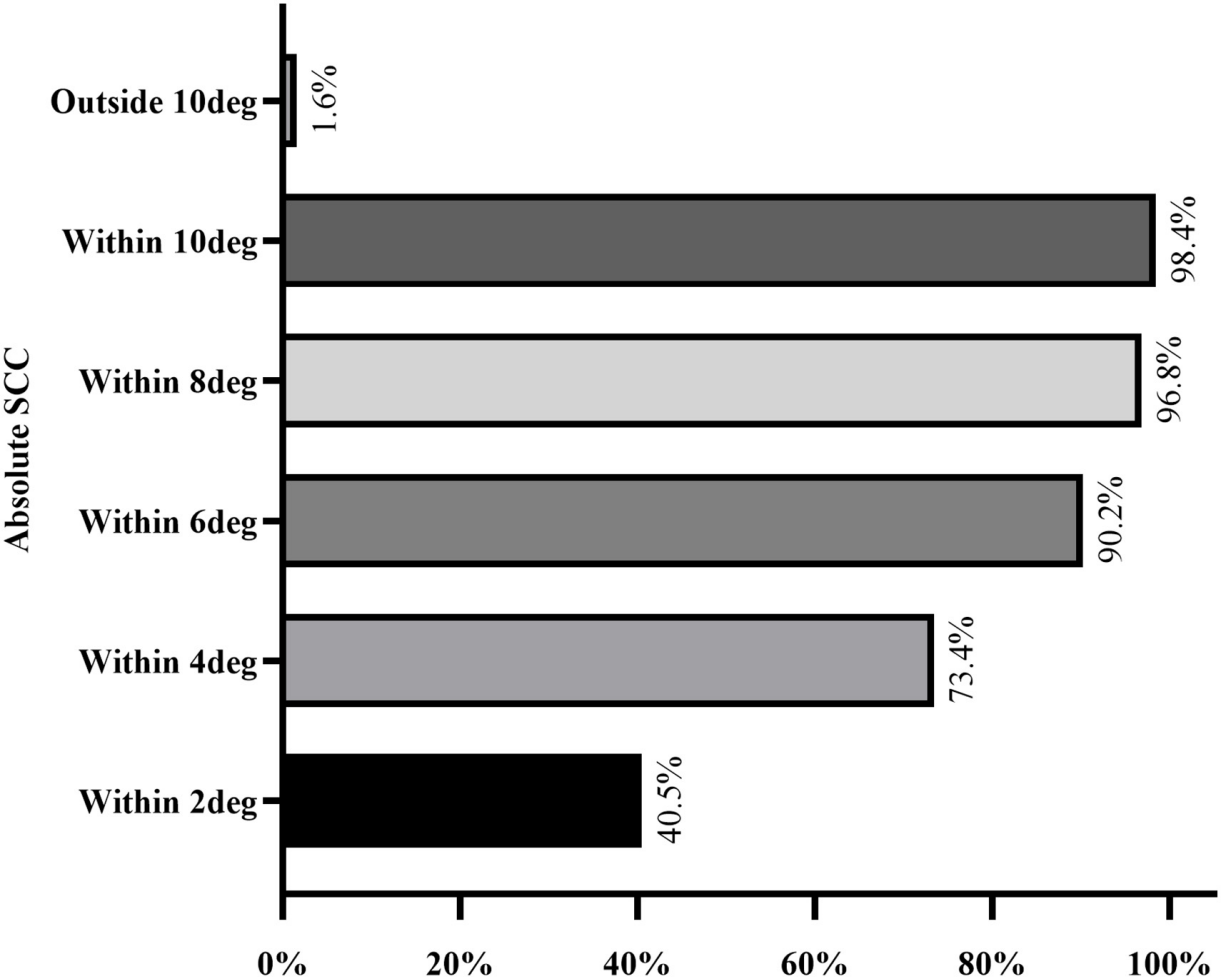
Distribution range of absolute SCC.

**FIGURE 2:**
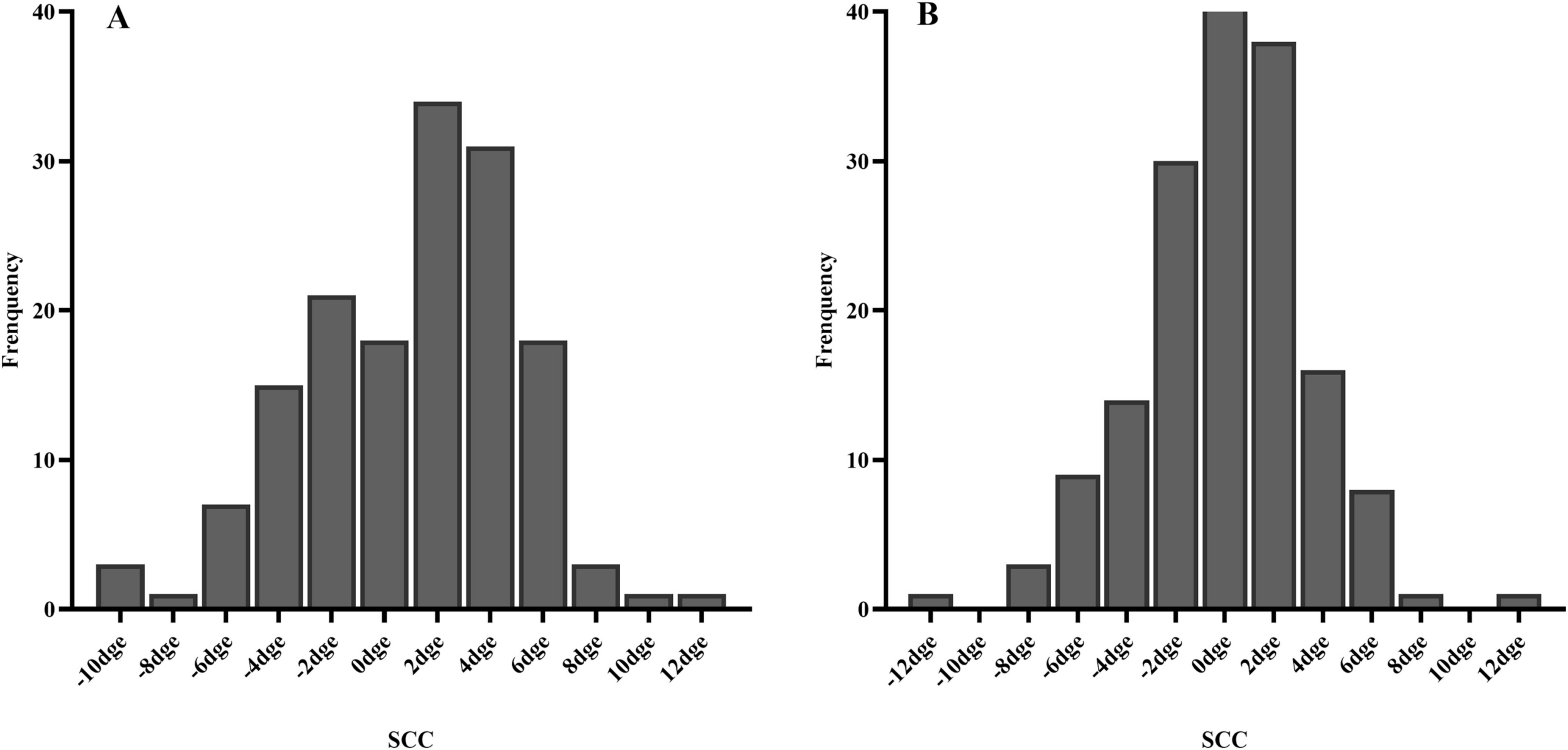
Histogram of SCC distribution **(A)** right eye; **(B)** left eye.

A total of 316 of 392 eyes (80.6%), including 153 right eyes and 163 left eyes, successfully registered static cyclotorsion before ablation. Multivariate logistic regression analysis showed that a thicker corneal flap (OR = 0.903, *p* < 0.01) was associated with a reduced risk of registration failure, whereas a higher laser cavity temperature (OR = 1.26, *p* = 0.047) was associated with an increased risk of static cyclotorsion registration failure (Table 2). The median absolute SCC among the 316 successfully registered eyes was 2.50 degrees (1.40–4.20 degrees) as shown in Table 3. Among them, 40.5% (128 eyes), 90.2% (285 eyes), and 98.4% (311 eyes) exhibited absolute static cyclotorsion within 2 degrees, 6 degrees, and 10 degrees, respectively. Additionally, 1.6% (5 eyes) exhibited static cyclotorsion greater than 10 degrees (Figure 1). The median SCC was 1.70 degrees (−1.82 to 3.80 degrees) in the right eye and 0.20 degrees (−2.07 to 1.97 degrees) in the left eye (Table 3). Figure 2 illustrates the histogram distribution of static cyclotorsion in both eyes. Among the right eyes, 95 (62.1%) exhibited static incyclotorsion and 56 (36.6%) showed static excyclotorsion. Among the left eyes, 76 (46.6%) showed static incyclotorsion and 85 (52.1%) showed static excyclotorsion. No statistically significant correlations were found between SCC and sex, age, laterality (right or left eye), refractive status, or intraoperative parameters (all *p* > 0.05).

**TABLE 2:** Multivariate logistic regression analysis of risk factors for static cyclotorsion registration failure.

Parameter	*B*	*P*	OR	OR (95% CI)
Sex (male = 1/female = 0)	−0.231	0.445	0.793	0.438–1.437
Age, years	−0.004	0.881	0.996	0.951–1.044
Eye (R = 1/L = 0)	0.382	0.156	1.465	0.865–2.48
Km, D	−0.144	0.165	0.866	0.706–1.061
Sphere, D	0.012	0.895	1.012	0.844–1.214
Astigmatism, D	−0.074	0.688	0.929	0.647–1.333
Astigmatism axis, degree	0.000	0.856	1	0.996–1.003
Flap thickness, μm	−0.102	0.007	0.903	0.839–0.973
CCT, μm	0.006	0.408	1.006	0.992–1.021
Temperature-mean,°C	0.232	0.047	1.261	1.003–1.584
Humidity-mean,%	−0.009	0.687	0.991	0.951–1.034
Pupil offset, μm	−0.001	0.552	0.999	0.996–1.002

Km, mean corneal keratometry; CCT, central corneal thickness; B, regression coefficient; OR, odds ratio; CL, confidence interval.

**TABLE 3:** Dynamic and static cyclotorsion parameters of eyes.

Parameter	Statistic	Total	*R*	*L*
*n*		316	153	163
SCC, degree	Median (Q1,Q3)	0.80 (−1.90, 3.00)	1.70 (−1.82, 3.80)	0.20 (−2.07, 1.97)
	Range	−12.2 to 11.5	−10.3 to 11.1	−12.2 to 11.5
Absolute SCC, degree	Median (Q1,Q3)	2.50 (1.40, 4.20)	3.10 (1.70, 4.72)	2.00 (1.00, 3.50)
	Range	0–12.2	0–11.1	0–12.2
*n*		387	193	194
Minimum DCC, degree	Median (Q1,Q3)	−0.43 (−0.72, −0.24)	−0.38 (−0.66, −0.21)	−0.47 (−0.82, −0.28)
	Range	−3.36 to 0.25	−3.36 to 0.25	−2.35 to 0.15
Maximum DCC, degree	Median (Q1,Q3)	0.52 (0.29, 0.80)	0.54 (0.31, 0.81)	0.51 (0.26, 0.80)
	Range	−0.08 to 4.36	−0.08 to 4.36	−0.01 to 2.83
Mean DCC, degree	Median (Q1,Q3)	0.04 (−0.15, 0.21)	0.06 (−0.12, 0.23)	0.01 (−0.19, 0.20)
	Range	−1.47 to 1.37	−1.09 to 1.05	−1.47 to 1.37
DCC amplitude, degree	Median (Q1,Q3)	1.00 (0.78,1.30)	0.98 (0.76,1.22)	1.02 (0.80,1.41)
	Range	0.38–6.44	0.40–6.44	0.38–3.36

SCC, static cyclotorsion component; DCC, dynamic cyclotorsion component.

**FIGURE 3:**
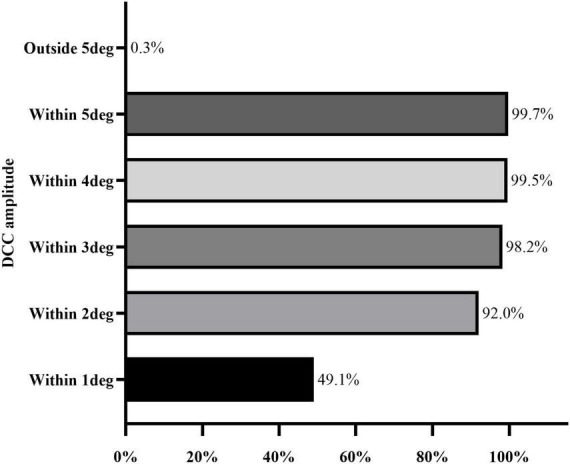
Distribution range of DCC amplitude.

**FIGURE 4:**
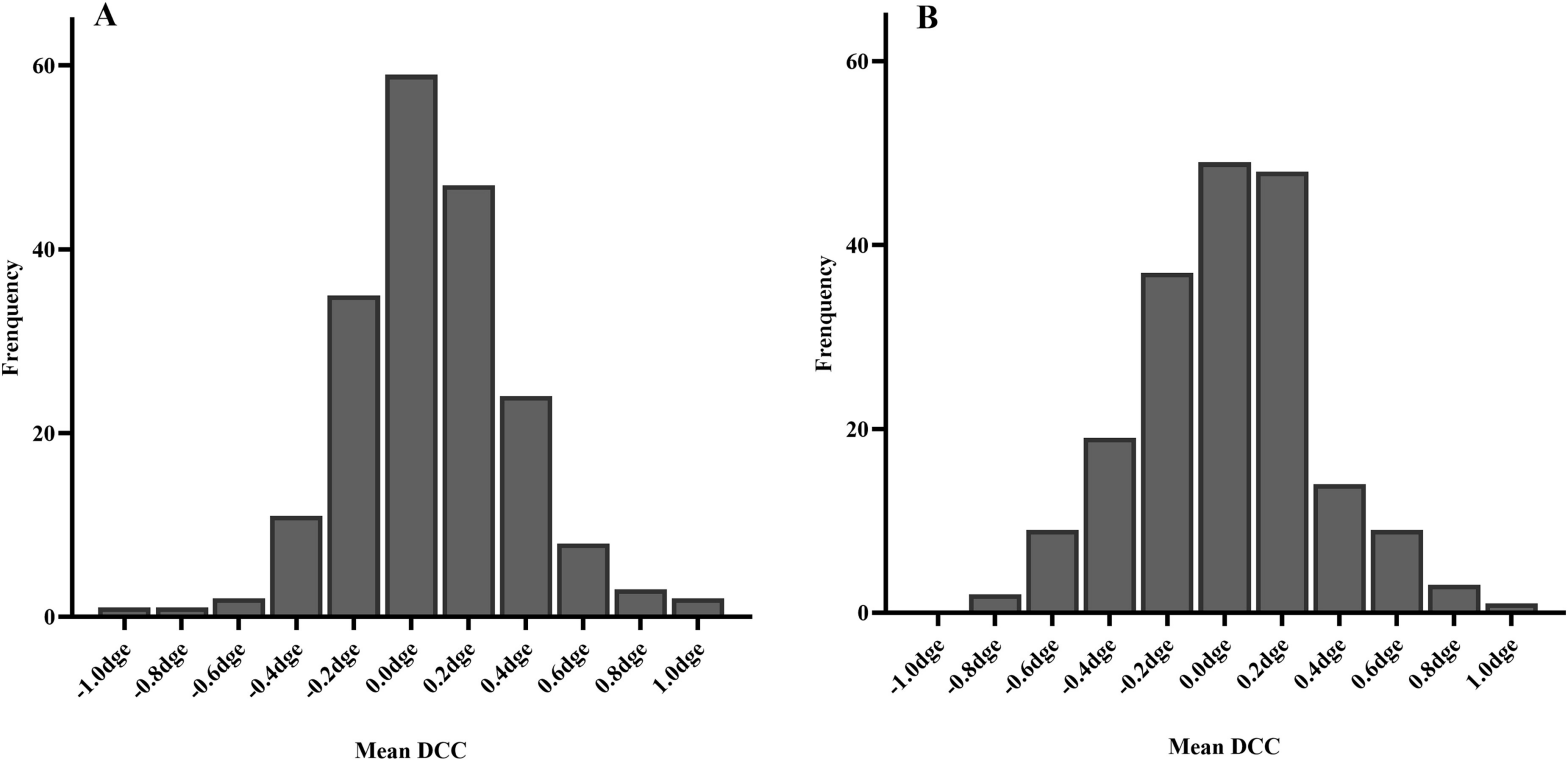
Histogram of mean DCC distribution **(A)** right eye; **(B)** left eye.

**FIGURE 5:**
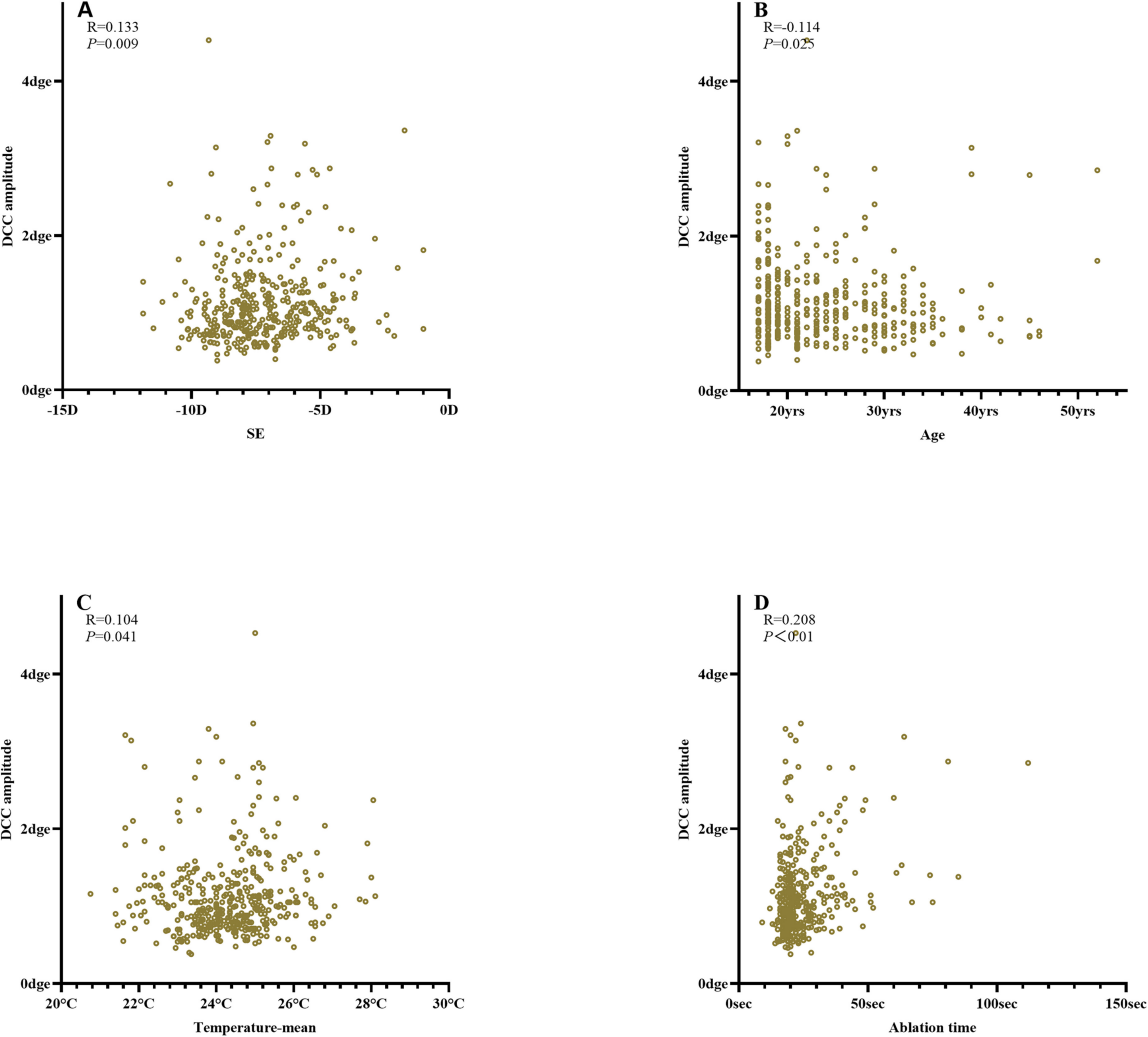
Correlation analysis of DCC amplitude **(A)** equivalent spherical power; **(B)** age; **(C)** laser cavity temperature; **(D)** ablation time.

The DCC registration success rate was 98.7% (387/392 eyes). The overall median DCC amplitude was 1.00 degrees (range: 0.78–1.30 degrees). Among the successfully registered eyes, DCC amplitudes were within 1 degrees, 3 degrees, and 5 degrees in 190 (49.1%), 380 (98.2%), and 386 (99.7%) eyes, respectively. One eye (0.3%) exhibited a DCC amplitude greater than 5 degrees (Figure 3). The mean DCC median was 0.06 degrees (−0.12 to 0.23 degrees) for the right eye and 0.01 degrees (−0.19 to 0.20 degrees) for the left eye (Table 3). The histogram distribution of mean DCC values for both eyes is shown in Figure 4. Dynamic incyclotorsion was observed in 116 right eyes (60.1%) and dynamic excyclotorsion in 73 right eyes (37.8%). Among the left eyes, dynamic incyclotorsion was observed in 94 (48.5%) and dynamic excyclotorsion in 98 (50.5%). Spearman’s correlation analysis revealed a negative correlation between age and DCC amplitude (*R* = −0.114, *p* = 0.025) (Figure 5B). Conversely, equivalent spherical power, laser cavity temperature, and actual ablation time were positively correlated with DCC amplitude (*R* = 0.133, *p* = 0.009; *R* = 0.104, *p* = 0.041; *R* = 0.208, *p* < 0.01) (Figures 5A,C,D). No statistically significant correlations were observed between sex, laterality, or other intraoperative parameters and DCC amplitude (all *p* > 0.05).

## 4 Discussion

As corneal refractive surgery advances toward a more personalized approach to improving visual quality, it is essential to ensure that data from comprehensive preoperative examinations accurately correspond to the actual laser ablation site during surgery. However, factors such as changes in patient position or anxiety can induce static or dynamic cyclotorsion, leading to deviations between the intended and actual ablation sites. Such discrepancies reduce correction accuracy and may compromise postoperative visual quality (2, 5–7, 9). Theoretically, rotational misalignments of 4 and 10 degrees can result in approximately 14 and 35% undercorrection of astigmatism, respectively (11). Studies have shown that combining static cyclotorsion compensation via iris registration with dynamic cyclotorsion tracking can improve refractive outcomes (12). The SCHWIND AMARIS excimer laser used in this study is designed to identify multiple ocular structures, including the pupil, limbus, and iris, while continuously monitoring eye rotation in real time to adjust the laser spot position accordingly (13).

Static cyclotorsion compensation determines the SCC angle by comparing characteristic points of the iris texture between the upright (pre-examination) and supine (surgical) positions (13). Laser ablation is then performed after compensating for this angle. However, achieving a high FS-LASIK static cyclotorsion registration success rate remains a clinical challenge. Registration failure impairs the system’s ability to compensate for static cyclotorsion, potentially leading to incomplete astigmatism correction. Our study identified thinner corneal flaps as an independent risk factor for SCC registration failure. This may be because of increased stromal resistance in thinner flaps, which restricts cavitation bubble movement and affects their dispersion into the surrounding tissue. The resulting microbubbles may interfere with the excimer laser’s ability to recognize iris texture (14, 15). Zhou et al. reported that 100 μm corneal flaps were more likely to exhibit an opaque bubble layer compared with 110 μm flaps (16). This phenomenon has also been observed in corneal caps of varying thicknesses during femtosecond small-incision lenticule extraction (SMILE) procedures (17). Similarly, in SMILE, the risk of developing an opaque bubble layer decreases as myopia increases, likely as a result of a deeper scanning plane (18).

In our study, the SCC iris recognition success rate was 80.6% lower than the 89.8% reported by Prakash et al. (4). This discrepancy may stem from differences in laser platforms and racial factors. The predominantly Asian cohort in our study likely had darker iris pigmentation, which reduces reflectance and complicates iris recognition (19). Additionally, elevated laser cavity temperature was associated with a higher risk of SCC identification failure. The underlying mechanism of this relationship remains unclear and warrants further investigation.

We also found that age (*R* = −0.114), equivalent spherical power (*R* = 0.133), laser cavity temperature (*R* = 0.104), and actual ablation time (*R* = 0.208) were correlated with DCC amplitude. Prakash et al. similarly reported that age (β = −0.16), pulse count (β = 0.39), and sex significantly affected DCC amplitude during LASIK, whereas eye laterality, flap creation method, and ablation mode did not (4). Although these findings are broadly consistent, the correlation coefficients observed in our study were smaller (*R* = 0.1–0.2), suggesting limited clinical relevance. Differences may also reflect variations in study design, equipment, or racial demographics.

Younger patients tended to exhibit greater DCC amplitudes. This may be related to higher intraoperative stress, reduced cooperation, and poorer fixation stability among younger individuals (20). Longer ablation times may further increase ocular fatigue, leading to greater dynamic cyclotorsion. Alipour et al. found that the number of laser pulses correlated positively with dynamic cyclotorsion and was directly related to procedure duration (4, 20, 21), consistent with our findings. High-frequency excimer laser systems may mitigate this effect by shortening ablation time. Although previous studies reported no significant association between spherical equivalent and DCC amplitude (3, 17), our results suggest that higher myopia is associated with smaller DCC amplitudes. The mechanism underlying this observation remains uncertain and requires further study.

This study has some limitations. Its retrospective design precludes establishing causal relationships, and the observed associations should therefore be interpreted cautiously. Additionally, postoperative visual outcomes, such as visual acuity, contrast sensitivity, refraction, higher-order aberrations, and irregular corneal astigmatism, were not analyzed. Future prospective studies with long-term follow-up are needed to clarify the clinical implications of static and dynamic cyclotorsion on postoperative visual performance.

In conclusion, a thin corneal flap and elevated laser cavity temperature were identified as risk factors for static cyclotorsion registration failure in FS-LASIK. Additionally, age, equivalent spherical power, laser cavity temperature, and actual ablation time were associated with dynamic cyclotorsion. Preoperative gaze-stability training may be beneficial for younger patients with high refractive errors to ensure smoother surgical procedures and improved accuracy.

## Data Availability

The raw data supporting the conclusions of this article will be made available by the authors, without undue reservation.
